# Growth-dependent recombinant product formation kinetics can be reproduced through engineering of glucose transport and is prone to phenotypic heterogeneity

**DOI:** 10.1186/s12934-019-1073-5

**Published:** 2019-02-02

**Authors:** Juan Carlos Fragoso-Jiménez, Jonathan Baert, Thai Minh Nguyen, Wenzheng Liu, Hosni Sassi, Frédéric Goormaghtigh, Laurence Van Melderen, Paul Gaytán, Georgina Hernández-Chávez, Alfredo Martinez, Frank Delvigne, Guillermo Gosset

**Affiliations:** 1 Departamento de Ingeniería Celular y Biocatálisis, Instituto de Biotecnología, Universidad Nacional Autónoma de México, Cuernavaca, Morelos Mexico; 2 Terra Research and Teaching Centre, Microbial Processes and Interactions (MiPI), Gembloux Agro-Bio Tech, University of Liège, Gembloux, Belgium; 3 Cellular and Molecular Microbiology (CM2), Faculté des Sciences, Université Libre de Bruxelles (ULB), Gosselies, Belgium

**Keywords:** Single cell, Metabolic engineering, Filamentation, Flow cytometry, Microfluidic imaging

## Abstract

**Background:**

*Escherichia coli W3110* and a group of six isogenic derivatives, each displaying distinct specific rates of glucose consumption were characterized to determine levels of GFP production and population heterogeneity. These strains have single or combinatory deletions in genes encoding phosphoenolpyruvate:sugar phosphotransferase system (PTS) permeases as PtsG and ManX, as well as common components EI, Hpr protein and EIIA, also the non-PTS *Mgl* galactose/glucose ABC transporter. They have been transformed for expressing GFP based on a *lac*-based expression vector, which is subject to bistability.

**Results:**

These strains displayed specific glucose consumption and growth rates ranging from 1.75 to 0.45 g/g h and 0.54 to 0.16 h^−1^, respectively. The rate of acetate production was strongly reduced in all mutant strains when compared with *W3110/pV21*. In bioreactor cultures, wild type *W3110/pV21* produced 50.51 mg/L GFP, whereas strains *WG/pV21* with inactive *PTS IICBGlc* and *WGM/pV21* with the additional inactivation of *PTS IIABMan* showed the highest titers of GFP, corresponding to 342 and 438 mg/L, respectively. Moreover, we showed experimentally that bistable expression systems, as *lac*-based ones, induce strong phenotypic segregation among microbial populations.

**Conclusions:**

We have demonstrated that reduction on glucose consumption rate in *E. coli* leads to an improvement of GFP production. Furthermore, from the perspective of phenotypic heterogeneity, we observed in this case that heterogeneous systems are also the ones leading to the highest performance. This observation suggests reconsidering the generally accepted proposition stating that phenotypic heterogeneity is generally unwanted in bioprocess applications.
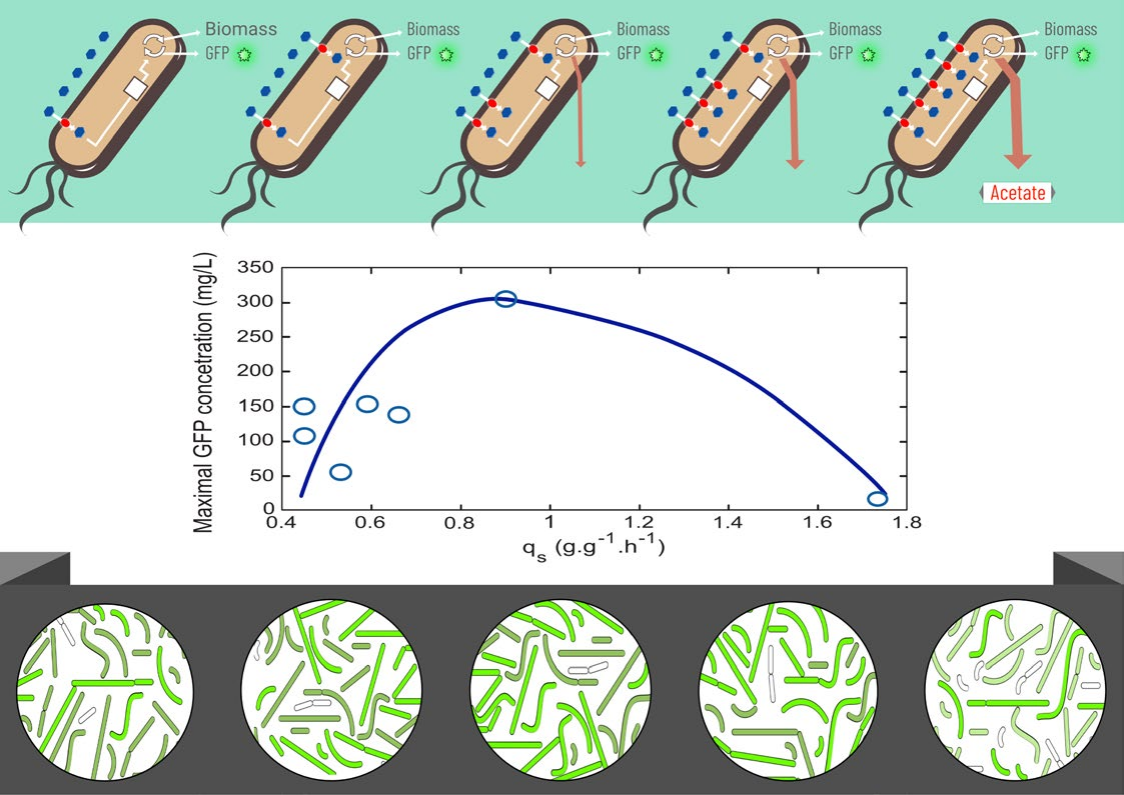

**Electronic supplementary material:**

The online version of this article (10.1186/s12934-019-1073-5) contains supplementary material, which is available to authorized users.

## Introduction

The bacterium *Escherichia coli* has been employed for the last 30 years as a host to produce recombinant proteins having medical applications. This organism has proven to be highly versatile, enabling the development of industrial production processes that have resulted in the successful placement of novel therapeutic proteins in the market. The cultivation of recombinant *E. coli* production strains commonly employs media containing glucose, since this carbohydrate is relatively inexpensive, and it is the preferred carbon and energy source for this bacterium. In *E. coli*, the entry of glucose into the periplasmic space is mainly dependent on the porin proteins *OmpC*, *OmpF* and *LamB* [1]. Once it is in the periplasm, glucose is internalized to the cytoplasm and phosphorylated by the phosphoenolpyruvate:sugar phosphotransferase system (PTS) [2]. Either when glucose is present in the medium at a very low concentration or when the PTS system is inactivated, other transport proteins such as the high-affinity ABC transporter *Mgl* system and the galactose:H^+^ symporter *GalP* contribute to its import. Under these conditions, the internalized glucose is phosphorylated by the enzyme glucokinase in an ATP-dependent reaction (Fig. 2) [3, 4].

**Fig. 1:**
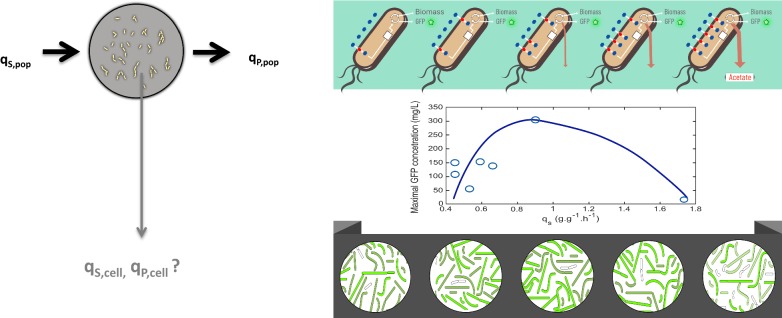
Outline of the methodology that has been considered in this study. The relationship between q_S_ and q_P_ exhibits a bell-shaped curve from experiments carried out in chemostat. Is it possible to reproduce this trend in batch bioreactors based on the utilization of strains with modified glucose import kinetics? For this purpose, q_S_ and q_P_ will be analyzed at the population level, but also at a single cell resolution based on flow cytometry and microfluidics

When *E. coli* grows aerobically in media where glucose is not limiting, it displays high specific rates of glucose consumption (*q*_*s*_) and growth (*μ*). It has been shown that under these conditions, an unbalanced metabolic state is generated, where the rate of acetyl-coenzyme A (*AcCoA*) synthesis surpasses its rate of consumption by the tricarboxylic acid cycle (TCA). The excess of *AcCoA* is diverted mainly into the phosphotransacetylase (*Pta*)-acetate kinase (*Ack*) pathway, resulting in acetate synthesis. The accumulation of acetate in culture media has a negative impact on strain productivity, since this organic acid is toxic for *E. coli* and it also represents the loss of the important metabolic precursor *AcCoA* [5, 6]. Strategies to eliminate or mitigate overflow metabolism include the use of glucose feeding strategies to limit the concentration of this sugar in culture media, as well as the generation of mutant strains with defective acetate-production pathways or reduced glucose import capacity [7–9]. The reduction of glucose import capacity by the inactivation of genes encoding glucose transporters has been proven a successful strategy for improving *E. coli* strains for the production of recombinant proteins, DNA vaccines and chemicals [10–12]. These reports and other examples show how the modification of glucose transport is a successful strategy to improve microbial cell factories. However, it is still not yet completely clear what are the consequences of such modifications on various aspects of cell physiology and how these responses translate to improved production characteristics. Previous studies carried out in similar context (i.e., production of recombinant β-lactamase, lymphokine, cyanase in *E. coli*) have shown that there is a significant growth-dependent recombinant protein formation kinetics [13]. Further experiments have shown that there is a bell-shaped curve describing the relationship between substrate uptake rate (qs) and recombinant product formation rate (qp) [13, 14] (Fig. 1). However, all the above-mentioned studies have been performed in chemostat, a cultivation operating mode which is quite hard to be applied at the industrial level. An important question that will be addressed in this study is to what extent strains with modified sugar uptake capabilities (i.e., qs) follow this bell-shaped trend.

The mechanistic reason behind this qs = f(qp) relationship seems to be attributable to growth dependent mechanisms, such as transcription and translation rates. However, it is known that these mechanisms are subjected to biological noise, leading to cell-to-cell difference at the level of qs and qp [15]. In order to address this issue, single-cell technology has attracted a lot of attention in recent years and has led to numerous fundamental results discussing the occurrence of phenotypic heterogeneity among clonal cell populations and the underlying mechanisms involved [16–18]. However, its impact on bioprocesses efficiency remains unclear, due to the lack of knowledge about the impact of phenotypic heterogeneity on bioprocess performance [19–21]. Indeed, most of the work addressing the issue of microbial phenotypic heterogeneity is related to nutrient assimilation and transition [22–24], but more rarely to the expression of heterologous proteins [25]. In this work, a group of seven isogenic *E. coli* strains including wild type and mutants were used (see Fig. 1 for a description of the experimental strategy). These strains where deleted for genes encoding *PTS* and non-*PTS* proteins involved in glucose transport and transformed with a *lac*-based expression plasmid expressing green fluorescent protein. Given that *lac*-based expression systems are subjected to bistability [26], this set of mutants was characterized in bioprocess condition using flow cytometry and microfluidics imaging. The impact of phenotypic heterogeneity on microbial performances was assessed by monitoring green fluorescent protein (GFP) accumulation rate. Significant cell-to-cell heterogeneity in GFP synthesis and cell elongation were observed with a clear correlation between the length of the filaments and the amount of GFP produced. Interestingly, we observed that the most heterogeneous system (represented by mutant *WG*-*pV21*) is also the one leading to the highest GFP production. This is a good case study for investigating the functionality of noise and related heterogeneity in a bioprocess perspective. Indeed, heterogeneity increases the fitness of microbial population in ecosystems but is generally unwanted for bioprocess application [25].

## Results and discussion

### Characterization of *E. coli* strains with reduced glucose import capacity: optimization of GFP production and limitation of by-products formation

**Fig. 2:**
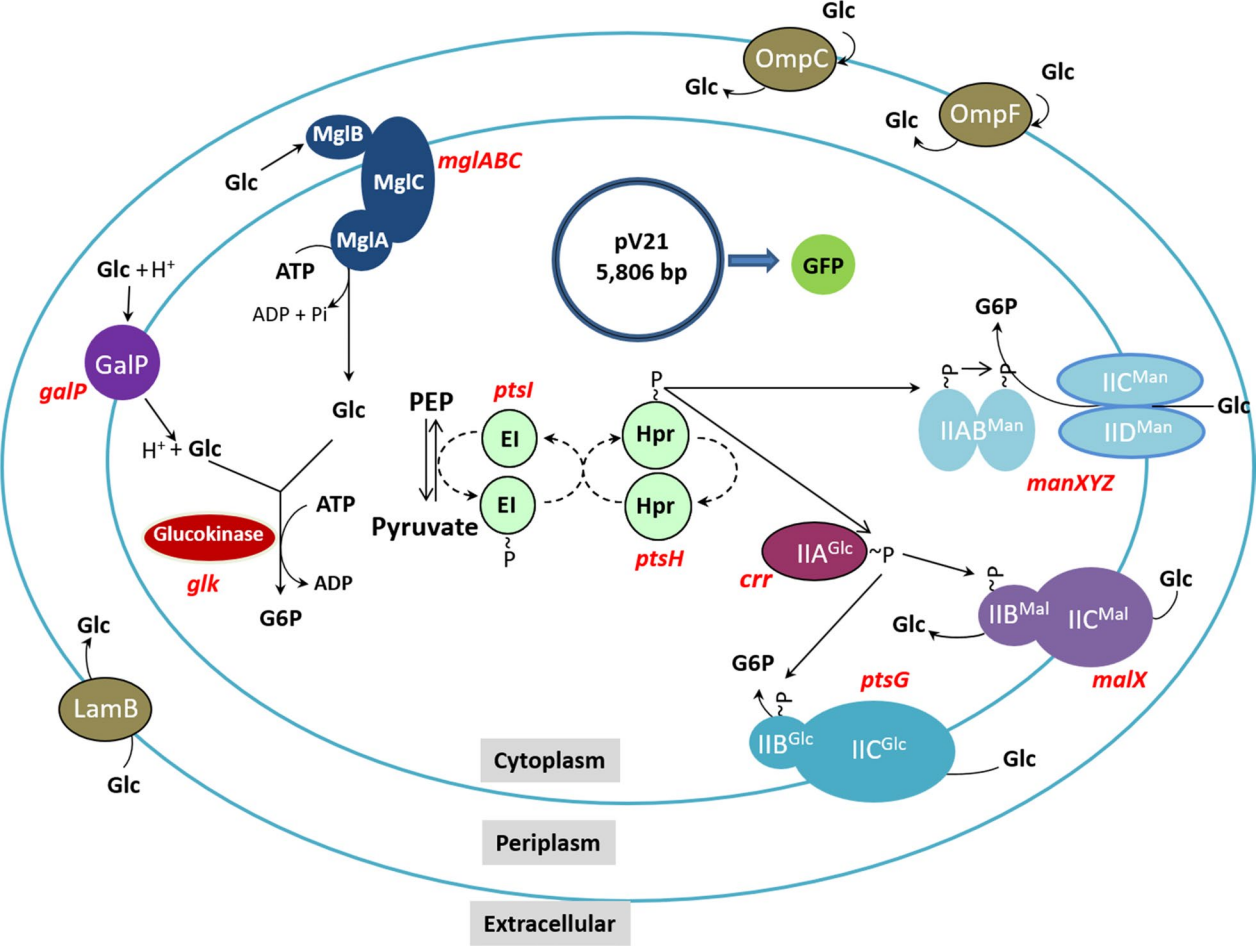
Proteins and enzymes involved in glucose transport and phosphorylation in *E. coli* and plasmid pV21 which encodes GFP. *EI*, enzyme I; *HPr*, phosphohistidine carrier protein; *IIA*^*Glc*^ and *IICB*^*Glc*^, components of the glucose PTS complex; *IIAB*^*Man*^ and *IICD*^*Man*^ components of the mannose PTS complex; IIBC^Mal^, components of the maltose PTS complex; *GalP*, galactose:H^+^ symporter; *MglA*, *MglB* and *MglC*, components of the galactose/glucose high-affinity ABC transporter; *LamB*, *OmpF* and *OmpC*, outer membrane proteins

**Fig. 3:**
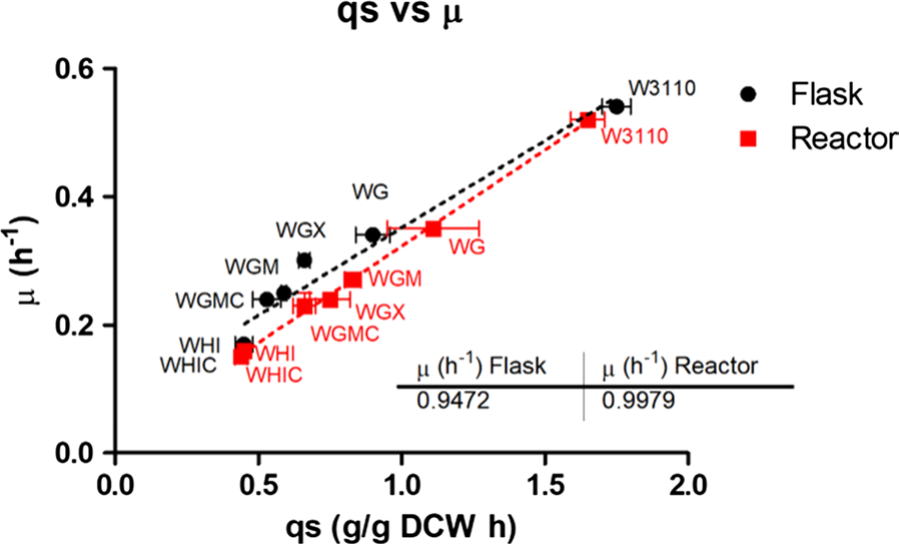
Specific growth rate as a function of the specific glucose consumption rate for the strain collection used in this study. Black circles and dotted line are showed the results from shake flasks, Red squares and dotted line are showed the result from stirred tank bioreactors

The *E. coli* strains employed in this study are part of a group of mutants having deletions of genes encoding PTS and non-PTS proteins involved in glucose import (Fig. 2). These strains showed a wide range of *q*_*s*_ values when growing in minimal salts medium with 2.5 g/L glucose [11]. From this group of mutants, six strains were selected that span the full range of observed *q*_*s*_ values (Table 1). Wild type *E. coli* W3110 and six mutant strains were transformed with plasmid *pV21*, carrying a gene encoding super glow GFP. The generated strains *W3110*/*pV21*, *WG*/*pV21*, *WGX*/*pV21*, *WGM*/*pV21*, *WGMC*/*pV21*, *WHI*/*pV21* and *WHIC*/*pV21* were characterized in shake flask cultures with minimal salts medium containing 10 g/L glucose as carbon source. The level of GFP was determined by measuring fluorescence as we mention below on “Materials and methods” section, this value reflects the active/soluble fraction of this protein. In Table 2 the kinetic and stoichiometric parameters of these cultures are summarized. The observed *q*_*s*_ and *μ* values under these conditions spanned a range of 1.75 to 0.45 g/g h and 0.54 to 0.16 h^−1^, respectively. A linear correlation between *q*_*s*_ and *μ* values was observed (R^2^ = 0.98) (Fig. 3). Cultures were stopped at the point of entry into the stationary phase at the times indicated in Table 2. At that time, residual glucose was detected in all cultures (Table 2). The specific rate of acetate production (*q*_*ac*_) for *W3110*/*pV21* was 0.47 g/g h with an acetate titer of 0.54 g/L after 10 h culture time. In contrast, all the mutant strains displayed much lower *q*_*ac*_ values. The final acetate titers for the mutant strains were lower when compared to *W3110*/*pV21*, except for *WGM*/*pV21* and *WGMC*/*pV21*, that displayed an unexpectedly high level of acetate accumulation. The amount of GFP varied widely among strains. In cultures with *W3110*/*pV21*, 11.94 mg/L of GFP accumulated after 10 h of culture time. In contrast, *WG*/*pV21* produced 305.41 mg/L of GFP in 14 h, being the strain displaying the highest GFP titer and productivity. The rest of the mutant strains accumulated GFP in a range spanning from 153.24 to 55.09 mg/L.

**Table 1:** *E. coli* strains and plasmid employed in this study

Name	Description	Source
Strains		
W3110	*E. coli* F-λ-*rph*-*1 IN(rrnD*-*rrnE)*1	[44]
WG	W3110 Δ*ptsG*::FRT	[11]
WGX	WG Δ*malX*::FRT	[11]
WGM	WG Δ*manX*::FRT	[11]
WGMC	WGM, Δ*mglABC*::FRT-Cm-FRT	[11]
WHI	W3110 Δ*ptsHIcrr*::FRT	[11]
WHIC	W3110 Δ*ptsHIcrr*::FRT-Cm-FRT Δ*mglABC*::FRT-Cm-FRT	[11]
Plasmids		
pV21	based on plasmid pBCSK+ pLac/*gfp, Sp*^*r*^	[9]
pJOQ	2001-bp multicopy plasmid, pBR322 origin of replication, pTrc/*gfp*-*His tag, Km*^r^,	[45]

**Table 2:** Kinetic and stoichiometric parameters of *E. coli* W3110 and derived glucose transport mutants expressing GFP in shake-flask cultures

Strain	*µ* (h^−1^)	*q*_*s*_ (g/g h)	*q*_*ac*_ (g/g h)	Max acetate (g/L)	Y X/S (g/g)	Max GFP (mg/L)	*q*_*GFP*_ (mg/g h)	Y GFP/X (mg/g)	Max biomass (g/L)	Culture time (h)
W3110/pV21	0.54 ± 0.01	1.75 ± 0.05	0.47 ± 0.03	0.54 ± 0.00	0.30 ± 0.02	11.9 ± 0.4	8.0 ± 0.5	7.7 ± 0.2	1.58 ± 0.04	10
WG/pV21	0.34 ± 0.00	0.90 ± 0.06	0.01 ± 0.00	0.06 ± 0.01	0.27 ± 0.01	305.4 ± 8.6	54.2 ± 1.3	211.5 ± 8.9	1.45 ± 0.03	14
WGX/pV21	0.30 ± 0.00	0.66 ± 0.02	0.04 ± 0.00	0.35 ± 0.03	0.27 ± 0.00	138.3 ± 25.9	19.5 ± 0.5	71.2 ± 18.9	1.94 ± 0.03	21
WGM/pV21	0.25 ± 0.00	0.59 ± 0.01	0.08 ± 0.00	1.06 ± 0.1	0.3 ± 0.00	153.2 ± 29.4	8.9 ± 0.1	83 ± 15.8	1.97 ± 0.03	21
WGMC/pV21	0.24 ± 0.00	0.53 ± 0.05	0.06 ± 0.01	1.19 ± 0.16	0.31 ± 0.01	55.1 ± 10.6	8.1 ± 0.1	27.5 ± 7.3	2.11 ± 0.07	21
WHI/pV21	0.17 ± 0.01	0.45 ± 0.03	0.004 ± 0.001	0.03 ± 0.01	0.29 ± 0.02	108.3 ± 22.9	13.6 ± 1.4	65.4 ± 15.8	1.93 ± 0.04	28
WHIC/pV21	0.16 ± 0.01	0.45 ± 0.02	ND^a^	0.00 ± 0.00	0.32 ± 0.02	150.7 ± 20.3	12.8 ± 2.2	97.6 ± 19.0	1.68 ± 0.15	32

Cultures were performed in M9 minimal salts medium with 10 g/L glucose

^a^Not detected

To characterize these strains under more controlled conditions, they were grown in stirred bioreactor employing minimal salts medium containing 20 g/L glucose. Under these conditions, the *q*_*s*_, *μ* and biomass yield from glucose (Y_X/S_) values were similar to those observed when growing with 10 g/L glucose, and *q*_*s*_ correlated linearly with *μ* (R^2^ = 0.997) (Table 2 and 3) (Fig. 3). The amount of accumulated acetate for strains *W3110*/*pV21*, *WG*/*pV21* and *WGX*/*pV21* were in the same range observed in shake flask cultures. In contrast, strains *WGM*/*pV21* and *WGMC*/*pV21* accumulated acetate at a level one order of magnitude lower than that observed with 10 g/L glucose. The higher production of acetate in shake flasks by these strains could be the result of oxygen limitation and the lack of pH control. In conditions were the oxygen level was maintained above 20% and pH at a constant value of 7.0, these strains displayed a much lower level of acetate accumulation. These results suggest that mutations in these two strains make them more sensitive to the stress conditions resulting from shake flask cultivation. The GFP titers in these cultures were higher for all strains when compared to growth in shake flasks. This can be explained considering that that more biomass was generated in the bioreactor cultures when compared to shake flask conditions. Strain *W3110/pV21* accumulated 50.51 mg/L of GFP, while all the mutant strains produced a higher titer, with strain *WGM/pV21* accumulating the highest GFP titer at 438.46 mg/L.

**Table 3:** Kinetic and stoichiometric parameters of *E. coli* W3110 and derived glucose transport mutants expressing GFP in stirred tank bioreactors

Strain	*µ* (h^−1^)	*q*_*s*_ (g/g h)	*q*_*ac*_ (g/g h)	Max acetate (g/L)	Y X/S (g/g)	Max GFP (mg/L)	*q*_*GFP*_ (mg/g h)	Y GFP/X (mg/g)	Max Biomass (g/L)	Culture time (h)
W3110/pV21	0.52 ± 0.01	1.65 ± 0.06	0.112 ± 0.064	0.49 ± 0.10	0.24 ± 0.01	50.5 ± 4.6	2.5 ± 0.1	10.5 ± 0.9	5.1 ± 0.4	12
WG/pV21	0.35 ± 0.00	1.11 ± 0.16	0.012 ± 0.003	0.26 ± 0.03	0.25 ± 0.01	342.0 ± 35.0	9.3 ± 1.7	71.8 ± 4.5	4.7 ± 0.4	22
WGM/pV21	0.27 ± 0.00	0.83 ± 0.03	0.005 ± 0.002	0.16 ± 0.02	0.26 ± 0.01	438.5 ± 6.6	12.8 ± 0.3	91.3 ± 0.7	4.8 ± 0.1	34
WGX/pV21	0.24 ± 0.01	0.75 ± 0.07	0.003 ± 0.000	0.15 ± 0.03	0.24 ± 0.01	231.9 ± 34.7	6.7 ± 0.9	49.2 ± 4.9	4.7 ± 0.3	26
WGMC/pV21	0.23 ± 0.02	0.66 ± 0.04	0.003 ± 0.000	0.17 ± 0.04	0.27 ± 0	252.8 ± 11.0	7.1 ± 0.8	52.2 ± 2.1	4.8 ± 0.1	35
WHI/pV21	0.15 ± 0.01	0.44 ± 0.02	0.008 ± 0.002	1.05 ± 0.01	0.22 ± 0.02	187.6 ± 44.2	3.1 ± 0.7	42.8 ± 9.6	4.3 ± 0.3	40
WHIC/pV21	0.16 ± 0.01	0.45 ± 0.00	0.011 ± 0.011	0.52 ± 0.06	0.25 ± 0.01	158.8 ± 21.3	2.5 ± 0.3	37.5 ± 5.6	4.3 ± 0.5	40

Cultures were performed in M9 minimal salts medium with 20 g/L glucose

These results show that mutants with *q*_*s*_ and *q*_*ac*_ lower than the wild type strain displayed higher GFP specific productivities and titers (Tables 2 and 3). Increased production of recombinant protein has been reported with others *E. coli* strains defective in glucose import [9, 27, 28]. However, in contrast to previous reports, in this study we characterized a group of strains displaying a wide range of *qs* values in order to characterize the potential correlation between glucose import capacity and recombinant protein production at the single-cell level. Under shake flask or bioreactor culture conditions, all mutants displayed lower rates of acetate production and higher GFP titers when compared to wild type strain. When considering the best producer strains, a 25- and 8.7-fold increase in GFP titers was observed under shake flask and bioreactor cultures, respectively. In both shake flask and bioreactor cultures, strain *W3110/pV21* displayed the highest *qs* values and relatively low GFP production rates. Strains with lower glucose import capacity showed increased GFP production, with a maximum *qGFP* observed near *qs* values of 0.8–0.9 g/g DCW h, corresponding to strain *WG/pV21* in shake flask and *WGM/pV21* in bioreactor cultures. As *qs* values decrease from 0.8–0.9 to 0.44 g/g DCW, a progressive reduction in *qGFP* is observed. This general trend indicated that strains displaying either very high or low *qs* values are not optimal for GFP production. These results show that for these strains and under these growth conditions, there is a *qs* value where GFP production is maximal, corresponding approximately to 50% of the value displayed by the wild type. Further characterization will be required to understand the cellular and population characteristics of GFP productive strains.

### Population segregation within productive strains: coexistence of high and low production states

**Fig. 4:**
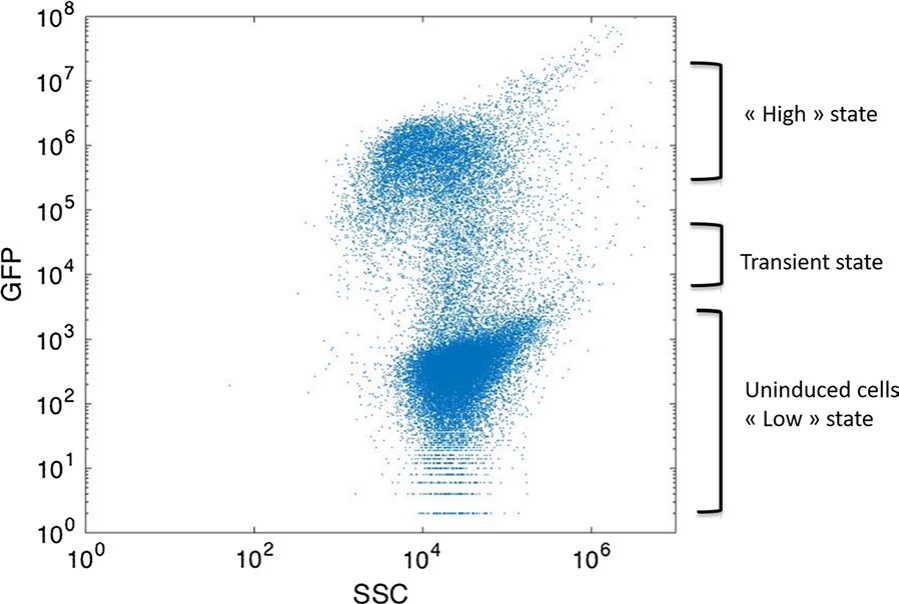
Example of population segregation occurring during cultivation of strain *E. coli WG* in bioreactor (sample collected after 6 h of culture)

We saw in the previous section that the three best GFP producer strains were *WG, WGX* and *WGM*. These strains reached almost a tenfold higher GFP accumulation in comparison with the *WT*. More precisely, from a single-cell perspective, after 4 h of culture, these strains appeared strongly segregated regarding GFP accumulation with the coexistence of “high” and “low” productivity physiological states (Fig. 4). In this regard, microbial segregation can be explained by the architecture of the *lac* operon.

**Fig. 5:**
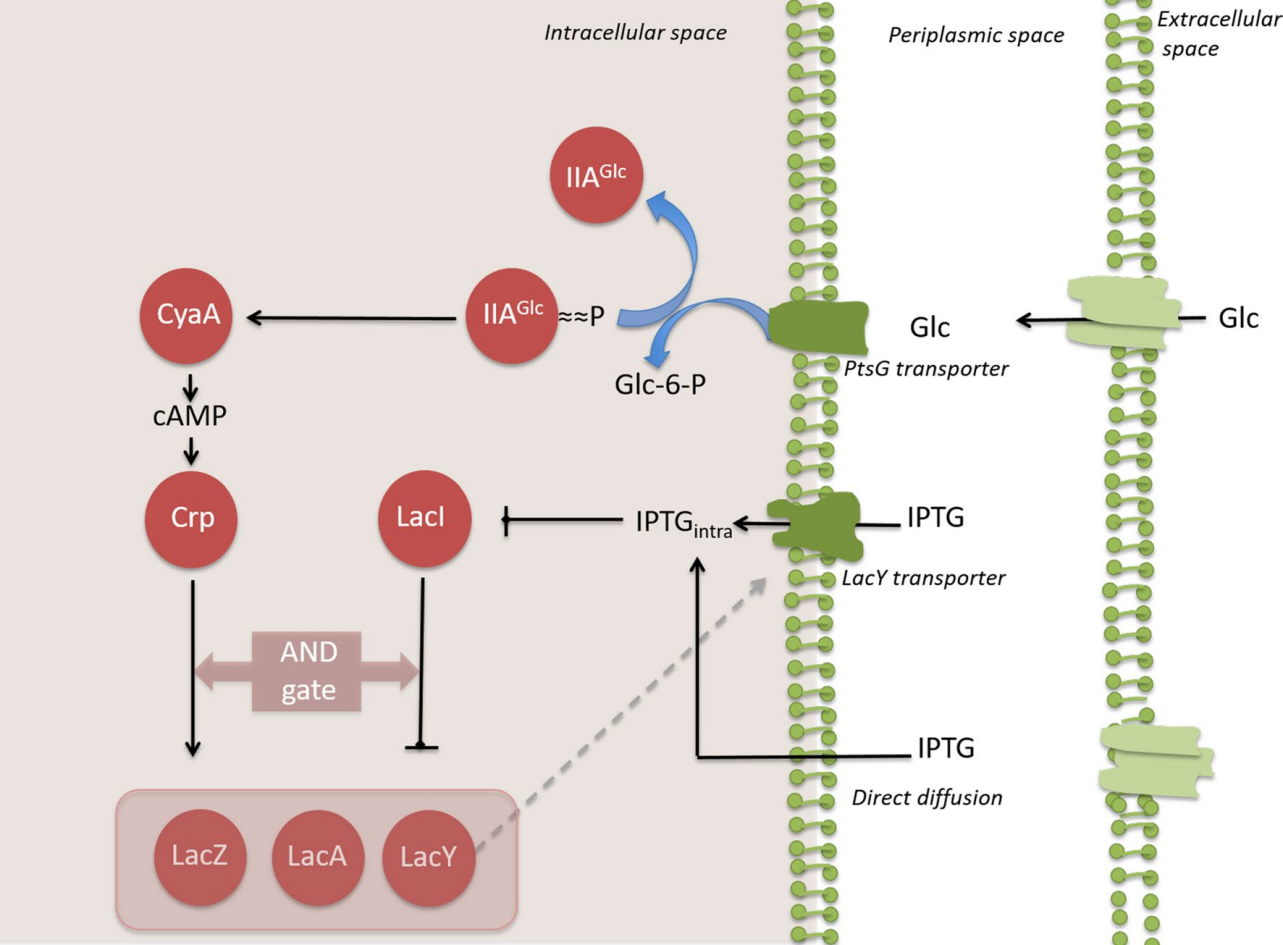
Scheme of the transcriptional regulatory network involved in the induction of the *lac* operon in *E. coli* (see text for detailed description)

Transcriptional network related to the *lac* operon is known to exhibit bistability [29] as most of the classical vectors used for production of recombinant proteins from *E. coli*. In the case of the *lac* operon, transcription is regulated by both the *crp* branch and the *lacI* branch [26]. The architecture of the *lac* operon ensures that the *lacZ*, *lacA* and *lacY* genes are activated only in presence of lactose and if glucose concentration is low. More precisely, if lactose is present in the medium, the constitutively expressed lactose repressor (*Lacl*) become inactive. Similarly, low concentration of intracellular glucose induces the phosphorylation of *IIAGlc*. Once phosphorylated, *IIAGlc* plays the role of an activator of adenylate cyclase. Consequently, cAMP intracellular abundance is inversely proportional to the intracellular glucose concentration. The complex formed by cAMP and *CRP* is an activator of the *lac* operon. In the conditions studied in this work, the intracellular glucose concentration is modulated by the deletion of glucose transporters. In that way, intracellular glucose concentration can be low even if medium contains high glucose concentration. This triggers the *crp* branch activation. In addition, the use of IPTG in the medium composition mimics the presence of lactose and activates the *lacI* branch. Thus, GFP production will be maximal if the glucose internal concentration is minimal and if the IPTG concentration is maximal. Of course, for evident metabolic reasons, a balanced intracellular concentration of glucose and IPTG must be considered (Fig. 5). In addition, Wurm et al. [30]. tested an *E. coli* BL21 strain using a PET expression vector to produce a recombinant protein (ScFv) using IPTG as inducer, they found that at low qs with a concomitant low µ the recombinant protein production is enhanced. They suggest that at low qs the catabolic repression effect is diminish and IPTG could be transported easily through lactose permease increasing the expression level. In this way, we showed that there is a *qs* value where the expression system induction is maximal, corresponding approximately to 50% of the value displayed by the wild type. Moreover, previous studies showed that even if the differentiation between “high” and “low” physiologic states is a bistable processes, once the state is differentiated, the switch to another state is a rare event. For this reason, deletion of major glucose transporter impacts on a long term the cellular dynamic [31, 32].

Moreover, biological noise has been previously described to impact microbiological performance [20, 23, 33]. For that reason, it appears interesting to investigate the physiology of the “high” productivity state. In that way, flow cytometry analysis provides single-cell patterns in order to depict biological noise during bioprocess [34]. However, this method does not allow to assess the single-cell differentiation between “high” and “low” productivity states. To bridge the gap of this technical limitation, real-time cellular imaging using microfluidic cultivation device will be addressed in the next section.

### High-production state characterization: the accumulation of GFP can trigger cellular filamentation

**Fig. 6:**
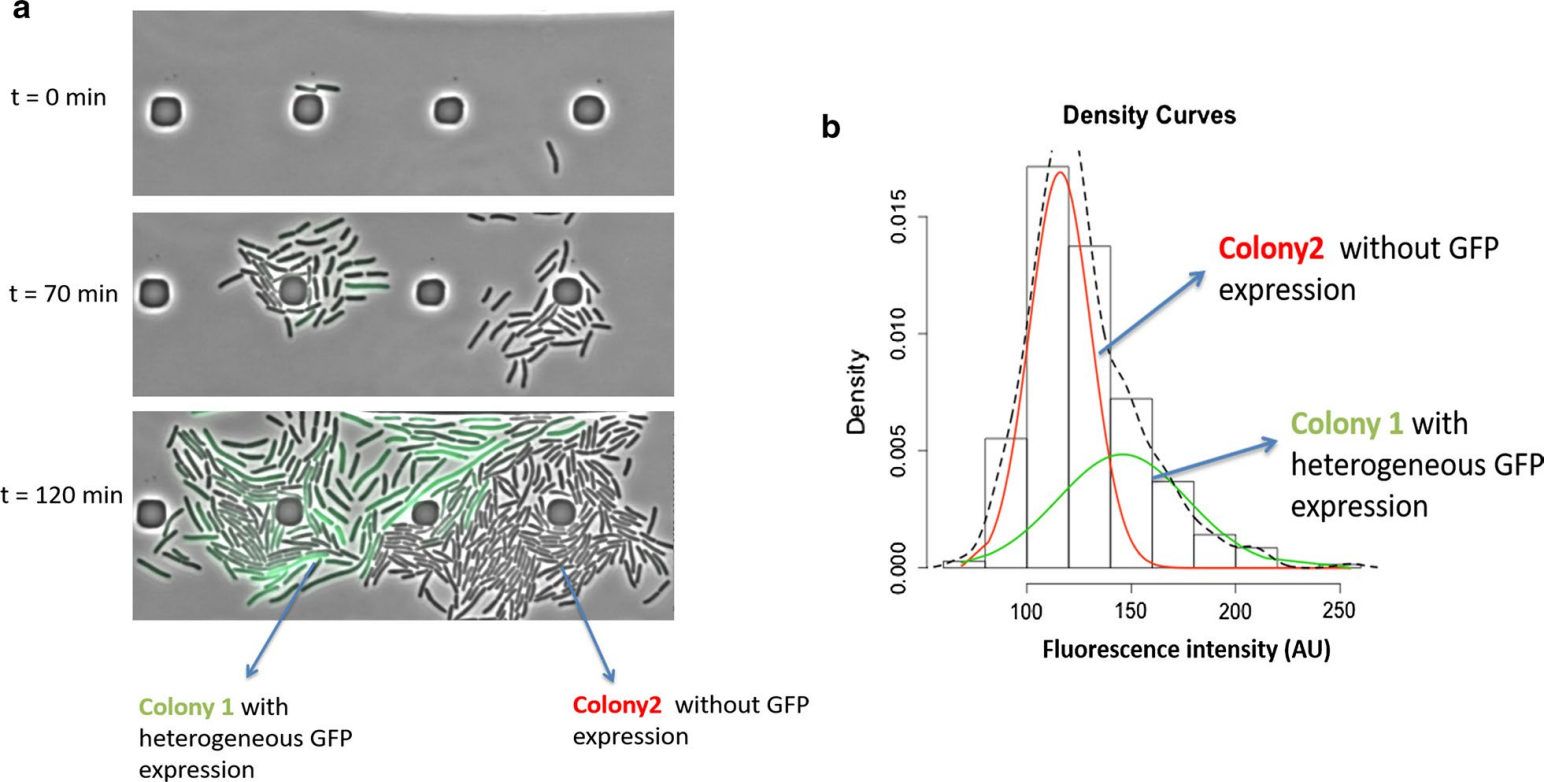
**a** Cultivation of *E. coli WG* in microfluidics. Image analysis after 120 min of culture (full movie is provided as Additional file 1). **b** Image analysis and deconvolution have been used to highlight subpopulations

Time-lapse cellular imaging using microfluidic cultivation device have been performed to track in real-time the lineage process behind GFP expression for the *WG* strain used in this study. Time-lapse pictures are represented on the Fig. 6a. After 120 min of cultivation, two major observations can be made. First, the two cells inoculated at t0 (t = 0 min on the Fig. 6a) provide two colonies with strong divergent physiologies since one (on the left) produced GFP and the other (on the right) did not produce GFP (Fig. 6a). This first observation has been addressed on a statistical basis to point out strong divergence in the physiological distribution among productive and non-productive colonies (Fig. 6b). Secondly, among GFP producing colony, a strong internal phenotypic heterogeneity is observed since only a fraction of the cells accumulates GFP. More precisely, it appears that cells producing GFP adopt also a filamentous morphology. Indeed, it has been shown previously that overexpression of recombinant protein can result in stress response and cell filamentation [35]). It can also be observed that filamentous cells are able to divide (see movie in Additional file 1), confirming the viability of these cells. Such “red-but-not-dead” phenotype has been previously reported [36, 37].

**Fig. 7:**
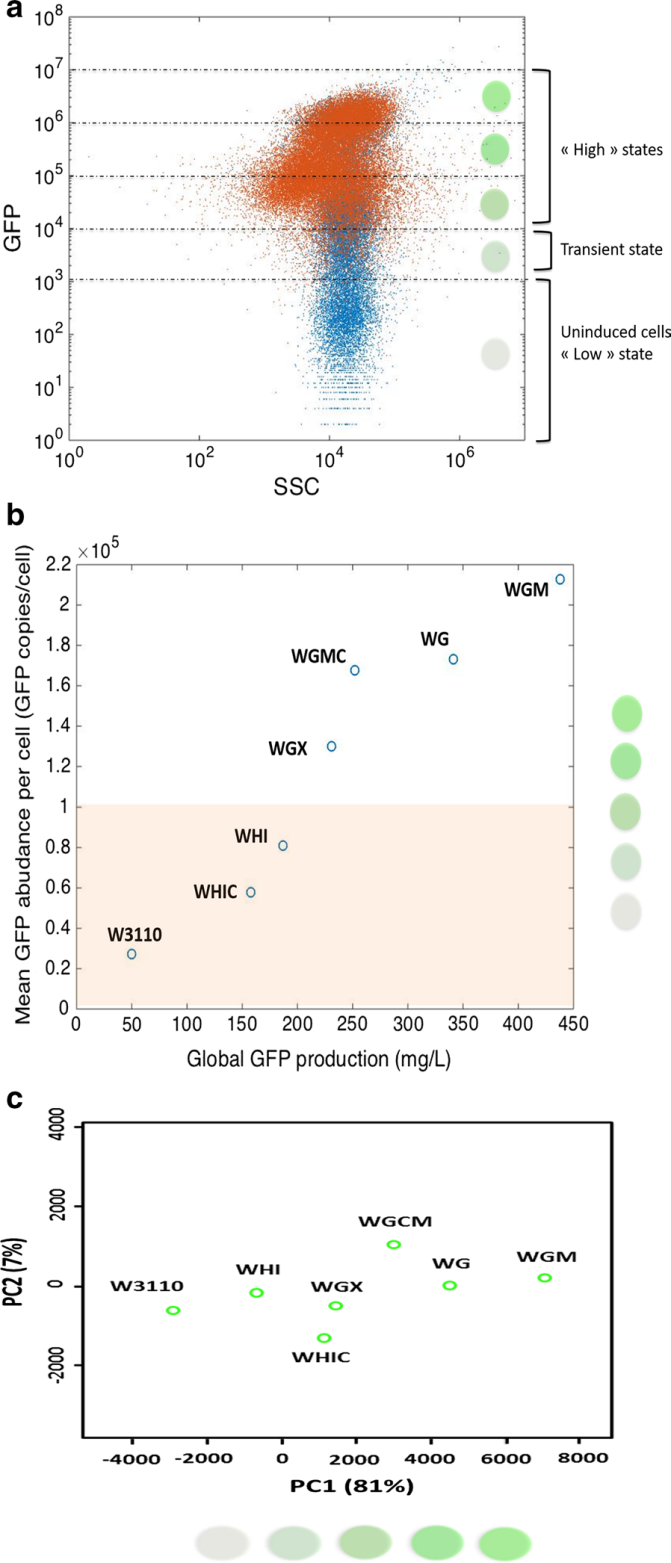
**a** Grid used for performing “physiological” fingerprinting. **b** Correlation between GFP production at the single-cell level and global production (orange shaded area corresponds to typical intracellular abundance for native proteins in *E. coli*). **c** Principal component analysis; the PCA plane represents the variability of strains regarding their two first principal components. PCA was performed on flow cytometry fingerprinting data. For this purpose, FL-1 versus. FSC cytogram were used for each mutant. Therefore, PCA gathers both morphological (FSC/filamentation) and physiological (FL-1/GFP accumulation) parameters

These results point out that GFP accumulation is paired with cellular filamentation. This observation can be interpreted regarding the strong accumulation of recombinant protein in the cytoplasm. Indeed, on a general basis, the total number of proteins for *WT E. coli* cells is about 3.10^6^ copies/cell. Among these proteins, the most abundant species are present up to 10^5^ copies/cell [38, 39]. However, flow cytometry analysis showed that for most of the mutants considered in this study, this value is largely exceeded to reach about 2.10^5^ copies/cell (Fig. 7). In that way, the intracellular GFP packing could crowd the cytoplasmic volume and reduce the diffusion of enzymes and their substrates within the cytoplasm that could impact others cellular function. In addition, previous studies demonstrated that it is possible to artificially trigger *E. coli* filamentation through inhibition of proteins involved in cellular septation such as *FtsZ* [40, 41]. Similarly, in this study, the filamentous phenotype could be triggered by the inactivation of enzymes involved in cellular septation. Additional observations showed that a local decrease of fluorescence within a filamentous cell is often associated with a localized cellular septation which corroborate this hypothesis.

**Fig. 8:**
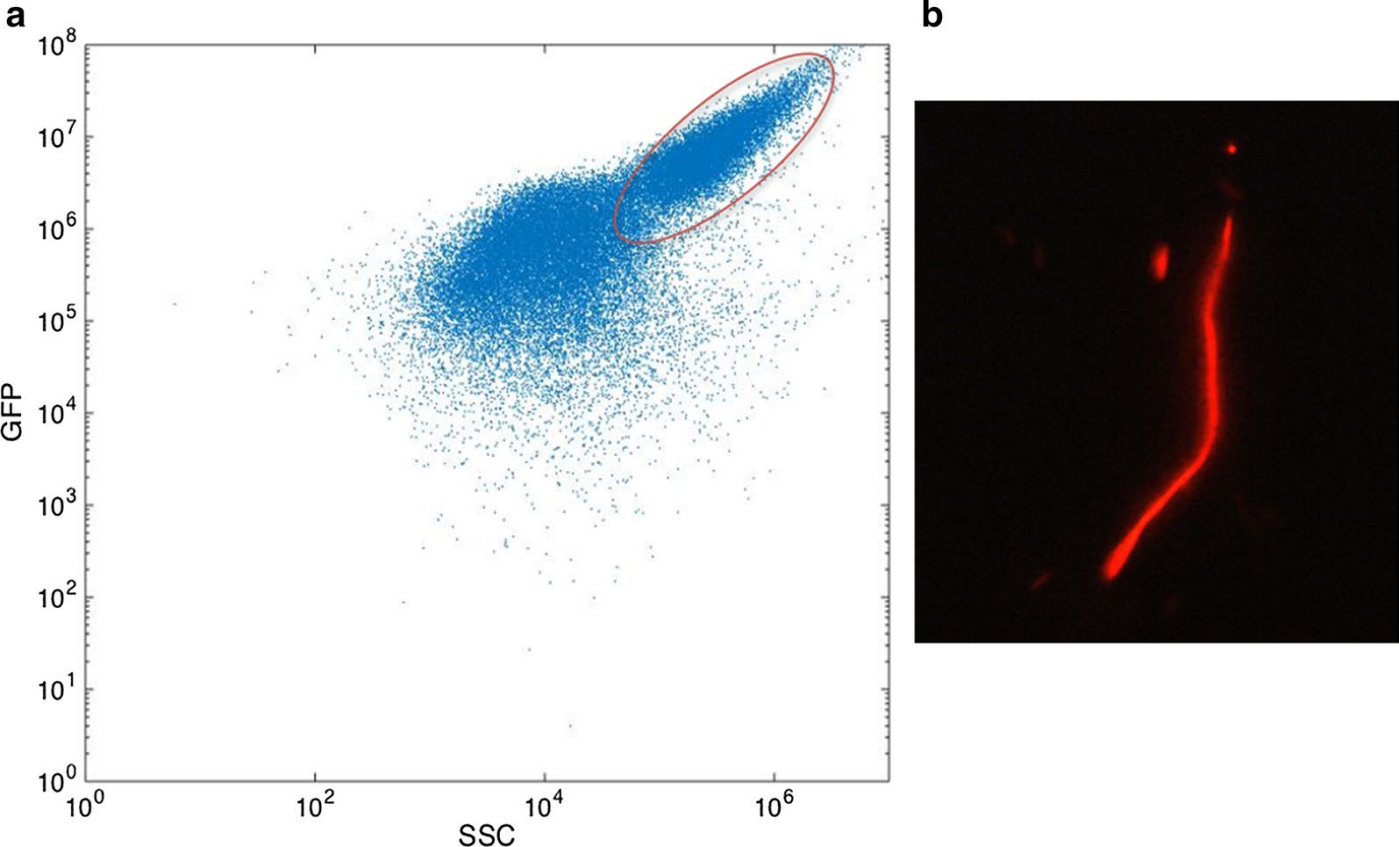
Flow cytometry analysis of the *E. coli WGM* strain after 12 h of cultivation in batch bioreactor. **a** Biparametric cell population distribution: x-axis represents the side scatter, y-axis represents intracellular GFP content (fluorescence units). **b** Fluorescent microscopy analysis revealed that filamentous cell fraction is delimited by the red ellipse. These cells exhibiting high affinity for PI

Moreover, the analysis of cytograms related with GFP best productive strain *WGM* (Fig. 8a) confirmed that the population is fully differentiated in the high state. Moreover, among this population, the most productive cells seem to display high side scatter channel (SSC) values (Fig. 8a). More precisely, we confirmed by fluorescent microscopy that high SSC sub-populations correspond to the filamentous fraction (Fig. 8b). The possibility to track both fluorescent and morphological traits supports the potentiality of flow cytometry to decrypt complex cellular attribute among microbial populations in routine analysis.

Finally, cell filaments appear to be strongly permeable to propidium iodide (PI) as shown in Fig. 8b, suggesting increased membrane permeability. PI has been commonly used as an exogenous biosensor to assess cellular viability since the molecule freely diffuses only through porous membranes to form a red fluorescent complex with DNA. In this case, filamentous cellular fraction appears as a “red-but-not-dead” phenotype [42] since these cells keep their ability to divide (see movie in Additional file 1). Moreover, increased membrane permeability leads to enhanced diffusion of IPTG, thereby inducing GFP synthesis. In that way, the previously observed correlation between morphological [Filamentation; Forward Scatter Channel/Side Scatter Channel (FSC/SSC) parameter] and physiological (GFP accumulation; FL-1 parameter) traits makes sense.

### Flow cytometry fingerprinting for monitoring biological performance

The results obtained so far clearly point out an impact of remodeling sugar transport on the efficiency of recombinant protein accumulation. However, this accumulation is accompanied by a strong phenotypic diversification of the microbial population, due to natural bistability of the *lac* system used for expressing GFP (Fig. 7a), but also due to morphological differentiation (Fig. 8). An interesting approach would be to merge both biological traits to ensure the correlation between biology and performance. However, this correlation is not straightforward considering the number of possible parameters that could be used for the characterization of phenotypic heterogeneity. The picture is even more complex since several time-points and several mutants should be considered. A specific methodology has thus been used to study, on a rational basis, the link between phenotypic heterogeneity and protein accumulation rate for the different strains used throughout this work. This methodology relies on the use of flow cytometry fingerprinting, a method originally developed for fast analysis of microbial communities [43].

The main advantage of flow cytometry fingerprinting is the possibility to characterize microbial populations based on their single-cell distribution patterns. Moreover, performing principal component analysis (PCA) based on FlowFP data allows to compare single-cell distribution on a statistical basis with a better resolution than considering only their mean or median value. This approach allows taking full benefit of the single-cell technology and is more relevant to assess biological performances in bioprocess conditions. In that way, this methodology has also potentialities for bioprocess monitoring using single-cell data. Moreover, it ensures that samples are comparable on a common basis and appear promising to manage efficiently the “big-data” challenge linked with single-cell techniques [20].

In this case, flow cytometry fingerprinting prior PCA (Fig. 7c) reveals that the distribution of mutants within the principal components follows the same trend than the one regarding their GFP productivity (Fig. 7b). In that way, FlowFP prior PCA strongly increases the robustness of the single-cell data analysis. However, as a major drawback, principal components don’t have any biological signification. This last point could compromise the outcome of this approach for bioprocess monitoring given that the loss of biological meaning can affect the possibility to determine how to regulate inefficient systems. For that reason, FlowFP and PCA appear promising but should be balanced regarding pros and cons of simpler alternative methods based on basic statistical analysis.

## Conclusions

We demonstrated in this study that it is possible to increase recombinant protein production rate through metabolic engineering of sugar import systems. Moreover, it has been shown that an artificial increase in microbial heterogeneity can be reached using bistable expression systems. More precisely, *lac*-*based* expression vector has led to strong population heterogeneity due to natural bistability. Therefore, in the conditions considered in this study, GFP producer strains exhibit coexisting “high” and “low” phenotypes regarding GFP accumulation. Flow cytometry analysis reveals that “high” state physiology exhibits a GFP accumulation beyond the natural threshold.

In addition, microfluidics imaging suggested that the major consequence regarding massive GFP accumulation could be an induction of cell filamentation. Additionally, filamentation is accompanied by an increase in membrane permeability, enhancing IPTG diffusion and consequently enhancing the high state physiology through the induction of *lac* promoter. However, the mechanism leading to this membrane porosity increase should be further investigated to understand which biological mechanism triggered it.

Moreover, single-cell analysis revealed that *WGM* and *WG* strains exhibit the best biological performances but are also the most heterogeneous systems regarding both GFP accumulation and cell morphology. This underlines the importance of finding appropriate metrics for the quantification of noise given that, in this study, microbial heterogeneity seems to be a robust proxy for microbial performance assessment. For this purpose, flow cytometry fingerprinting analysis prior principal component analysis constitutes a robust approach for monitoring both morphological (FSC/filamentation) and physiological (FL-1/GFP accumulation) parameters. Previous studies carried out in chemostat have shown significant growth-dependent recombinant protein formation kinetics. In the present study, we have shown that we can reproduce this bell-shaped dependency by using a set of mutants with modified glucose import capabilities. This approach is more convenient since mutants can be cultivated in batch bioreactor, further facilitating bioprocess up-scaling. Additionally, the proposed mutants exhibit decreased overflow metabolism and are then more robust to substrate fluctuations experienced in large-scale bioreactors. We have also shown that cell-to-cell heterogeneity could be a key player potentially explaining the dynamics of GFP production.

Finally, from a more fundamental perspective, the fact that the most performant systems are also the more heterogeneous is in opposite to the paradigm stating that only homogenous population are attractive for bioprocess applications. However, this observation has been made in ideal operational conditions given that bioreactor was considered as perfectly mixed and homogenous during the whole batch cultivation.

## Materials and methods

### Microbial strains

The strains used in this work are described in Table 1. The reference strain *E. coli* W3110 is a derivative of K-12 [44]. Strains WG, WGX, WGM, WGMC, WHI and WHIC are derivatives of W3110 [11]. The pV21 plasmid was based on plasmid pBCSK+ from Stratagene (La Jolla, CA). It contains the Superglo GFP gene (*gfp*) placed under transcriptional control of the promoter of the *lacZ* gene and a gene conferring spectinomycin resistance (Spc) [9]. The transformants W3110/pV21, WG/pV21, WGX/pV21, WGM/pV21, WGMC/pV21, WHI/pV21 and WHIC/pV21 were obtained by electroporation, stored in 40% glycerol solution and kept frozen at − 70 °C.

### Growth media and cultivation conditions

For shake flasks and stirred tank bioreactors cultures, we used M9 mineral medium containing 10 or 20 g/L glucose, 0.05 or 0.15 mL of trace elements solution, respectively. The M9 medium contained 6 g/L Na_2_HPO_4_, 3 g/L KH_2_PO_4_, 0.5 g/L NaCl, 1 g/L NH_4_Cl, 0.5 g/L MgSO_4_, 0.01 g/L CaCl_2_, 0.01 g/L thiamine hydrochloride, 30 mg/L Spc, 0.1 mM IPTG. The trace elements solution contained the following: 1.5 g/L Na_2_EDTA·2H_2_0, 0.45 g/L ZnSO_4_·7H_2_0, 0.03 g/L MnCl_2_·4H_2_0, 0.1 g/L H_3_BO_3_, 0.04 g/L Na_2_MoO_4_·2H_2_0, 0.3 g/L FeSO_4_·7H_2_0, and 0.03 g/L CuSO_4_·5H_2_0. Glucose and salt solutions were sterilized separately at 121 °C for 20 min, Spc, IPTG, thiamine, and trace elements solutions were sterilized by filtration and kept frozen at − 20 °C. The inoculum for shake flasks and stirred tank bioreactor cultures consisted of 5 mL overnight cultures in Luria–Bertani (LB) medium with 30 mg/L Spc, that were added to shake flasks containing 50 mL of M9 medium with 10 or 20 g/L glucose starting at an OD_600nm_ = 0.1 and incubated at 37 °C, 300 rpm in an orbital shaker until an OD of 2.0 was reached. Then, a sample was used to inoculate either shake flasks or stirred tank bioreactors containing 50 or 150 mL of the same medium, starting at an OD_600nm_ = 0.1. Shake flasks cultures were performed at 300 rpm and 37 °C. Batch cultures in stirred tank bioreactor were performed in a Dasgip mini-bioreactor platform (DASGIP DASbox Reactor SR0250ODLS). The reactors were equipped with controls for pH, temperature, agitation, and dissolved oxygen. NH_4_OH and H_3_PO_4_ solutions were automatically added to control pH at 7.0. Temperature was maintained at 37 °C, dissolved oxygen was manually maintained above 20% by changing stirring speed, and airflow was set to 100 mL/min. Samples were taken periodically for offline analyses. All cultures were performed in triplicate.

### GFP protein purification

Superglo GFP protein expressed in *E. coli* strain MC1061 transformed with the constitutive non-commercial pJOQ plasmid [45] was produced in 200 mL of LB supplemented with kamamycin at 25 μg/mL, growing the culture at 37 °C for 20 h, under agitation at 200 rpm. After this process, the cells were recovered by centrifugation, re-suspended in 20 mL of PBS and lysed by sonication in three pulses of 2 min, with intermediate cooling on an ice-water mixture for 10 min. After centrifugation at 13,000 rpm, the clear supernatant was loaded on a HisTrap HP/1 mL column (GE Healthcare), and the protein was purified using an imidazole gradient from 30 mM to 300 mM in 30 min, at a flow rate of 0.75 mL/min, with detection at 280 nm. Imidazole solutions were prepared in 100 mM phosphate buffer (pH 7.2) containing 0.5 M NaCl. Fractions containing the pure protein were combined, concentrated on centrifugal filter devices (Amicon Ultra-4 10k, Millipore) and washed with PBS (2 × 4 mL) to remove the residual imidazole. The pure protein was re-dissolved in 1.5 mL of PBS and its concentration was determined by the Bradford assay using BSA standards; absorbance was measured using a DU 730 UV/Vis spectrophotometer (Beckman Coulter). The purity of the protein analyzed by denaturing SDS-PAGE and Coomassie staining was found to be greater than 95%.

### Analytical methods

Cell concentration was determined by spectrophotometry at 600 nm. The employed OD_600nm_ and cell dry weight (CDW) (g/L) correlation was CDM = 0.37 × OD_600nm_ [46]. Samples were diluted with phosphate buffer (PBS pH = 7) to reach an absorbance spanning from 0.1 to 0.8. Samples were centrifuged, and the supernatants were analyzed to determine glucose and acetate concentrations employing an Aminex HPX-87H column (300 × 7.8 mm; 9 Am Bio-Rad, Hercules, CA). Separation was carried out isocratically with 5 mM H_2_SO_4_ at flow rate of 0.5 mL/min and a temperature of 50 °C. Glucose was detected by refraction index and acetate by photodiode array at 210 nm. For these measurements, a Waters HPLC system was used: 600E quaternary pump, 717 automatic injector, 2410 refraction index, and 996 photodiode array. Also, biomass from shake flasks cultures were used to measure GFP production, it was detected by fluorescence readings in a Perkin Elmer LS55 Luminescence spectrometer. Wavelengths of 480 nm and 510 nm were used for excitation and emission respectively. Samples were diluted with PBS when needed to obtain readings within the linear range [9]. We used the GFP purified as a standard of concentration and fluorescence.

### Flow cytometry analysis

**Fig. 9:**
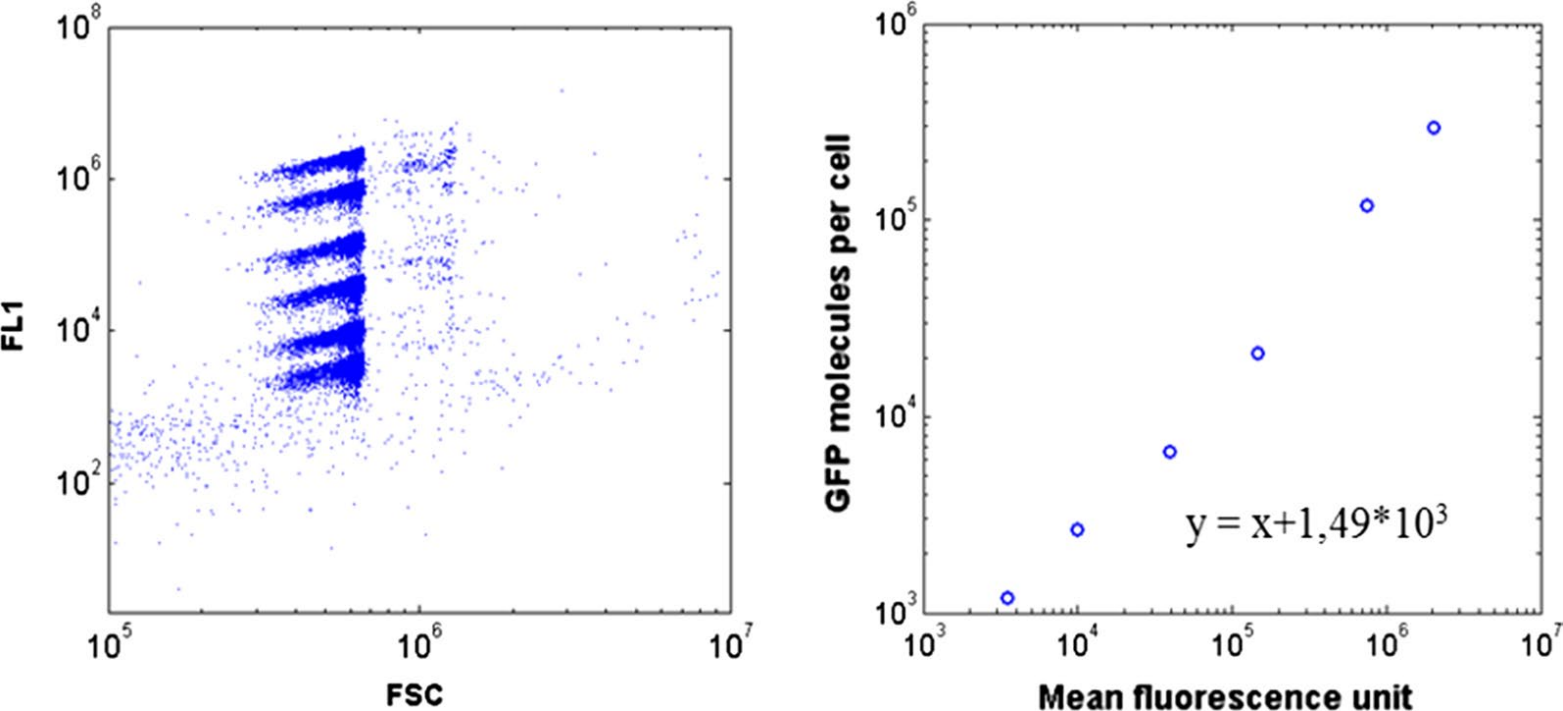
(Left) cytogram of the validation beads. The 6 spots correspond to 6 bead populations that have different specific amount of coated GFP molecules. (Right) Mathematical regression that links FL-1 fluorescence intensity with an amount of GFP molecules

Biomass culture samples from the Dasgip mini bioreactor were diluted in PBS to fit into the desired range of 500–2500 events/µL for cytometry analysis. Propidium iodide (PI) (Sigma Aldrich Fluka, Saint-Louis Missouri USA) was added to flow cytometry samples at a final concentration of 5 µg/mL. Flow cytometry was performed by analyzing 40,000 events (forward scatter threshold > 80,000; sheath fluid flow rate set to medium) using a C6 Accury Flow Cytometer (BD Biosciences, NJ, USA). GFP production was measured on the FL1 channel and PI staining on the FL3 channel. Raw data were extracted as fcs files and loaded into MATLAB by using the readfsc function (by L. Balkay, University of Debrecen, Hungary, available on MATLAB central file sharing). Cells were excited at 488 nm. FL1 and FL3 channels were processed to compute the mean and standard deviation (SD) of GFP and PI staining intensity at the level of the bacterial population [47, 48]. Additionally, FL-1 signal intensity was converted into GFP intracellular concentration. For this purpose, polystyrene beads coated with a given number of GFP molecules (Clontech, USA) were analyzed by flow cytometry. We were able to observe six distinct beads populations displaying specific fluorescence intensities (Fig. 9). Fluorescence data were extracted into MATLAB to compute the correlation between FL-1 signal and the corresponding amount of GFP molecules.

### Flow cytometry fingerprinting and principal component analysis

The raw data are extracted from the CFlow software (Accuri, BD Bioscience) as.fcs files and loaded into R software. Data analysis pipeline is adapted from the version developed by De Roy et al. [49] and consists in two principal steps. (i) Creating a n-dimensional quantitative fingerprint of each sample from their respective bivariate (SSC–FL1) flow cytometry (FCM) distribution using the recursive probability binning (PB) algorithm for flow cytometry data, implemented in the Bioconductor package FlowFP [50]. In a first time, a model, composed of hyper-rectangular regions (bins) of varying size and shape, is established. From superimposed FCM distribution (i.e. data from all samples are pooled together) and thanks to Probability Binning (PB) algorithm [51], bivariate data space is divided in hyper-rectangular regions in such way that each contains similar number of events (one event corresponding to one cell). First step of the algorithm consists in the division of space into two bins containing similar number of events. Afterwards, each of these bins is again divided into two bins with equal number of events, and so forth. Therefore, region of bivariate FCM space displaying high density of events are characterized by bins of small area whereas larger bins characterized regions of weak density. Moreover, the final number of bins (n*) is arbitrary set and correspond to 2^i^ with i the number of recursive subdivisions. In our case, 6 recursive subdivisions are applied and therefore the model is composed of 64 bins. The obtained model is then applied to each sample and number of cells per bin is determined, creating feature vector of counts (n*-dimensional) for each sample. The latter will be used as a fingerprint of the microbial community at a given time and under given conditions. (ii) Principal component analysis of the different fingerprints. The 64 bins are considered as the variables described for each sample by the number of cells per bin determined previously the data treatment has been previously described by Kinet [52].

### Real time cellular imaging in microfluidic cultivation device

The microfluidic cultivation device was the CellASIC^®^ ONIX B04A-03 Microfluidic Plate (Millipore, USA). This is a 4-chamber cell culture plate designed for use with the CellASIC^®^ ONIX Microfluidic System. The B04A plate has 4 independent units, each with 5 inlet wells, a cell inlet, a cell outlet, and a large outlet well. Each row of wells addresses the corresponding culture chamber and the plate is shipped preprimed with a PBS solution. The culture chamber dimensions are 2.0 × 1.2 mm in area with trap heights of 0.7, 0.9, 1.1, 1.3, 2.3 and 4.5 μm respectively. The microfabricated chamber gently holds cells against the glass imaging surface to maintain a single focal plane during perfusion-based imaging experiments. Before each microfluidic cultivation experiment, a precultivation step was performed in 100 mL of the medium mentioned before in baffled shake flasks at 37 °C and under orbital shaking at 160 revolutions per minute. Baffled flasks were inoculated with a single colony coming from a petri dish. Once cells reached mid-exponential phase, biomass was centrifuged washed two times in PBS and finally resuspended in fresh medium. Dilutions were then made in the same medium to a final OD600 0.03. A 50 μL aliquot of the final solution was injected in the inoculation well of a CellASIC ONIX microfluidic plate B04. Cells are injected in a culture chamber in which they are trapped and grow in a single focal plane. In this chamber, bacteria are under continuous perfusion of culture medium due to the 5 inlets. Detailed protocols for the cell-loading and cell-culture can be found on the user guide provides by the supplier. Image acquisition was performed at 30 min intervals. Temperature was set at 37 °C during the time of the experiment and the microfluidic chamber was put above a 100× oil immersion objective on an inverted microscope (Axio Observer, Zeiss, Germany). Illumination was provided using an HXP lamp (120 V). For fluorescence measurements, 38 HE filter set was used (Zeiss, Germany). Fluorescence was recorded by a Hamamatsu ORCA-Flash 4.0 digital camera. Image analysis (including cell segmentation, classification and deconvolution of the subpopulations) was performed with the freeware Fiji53, using the MicrobeJ plugin54 and with MicrobeTracker55 in MATLAB (R2013a, Mathworks), assisted by custom-made scripts.

## Additional file

**Additional file 1.** Time-lapse movie of microfluidic experiments involving strain WG. In this movie, a specific focus has been made on a filamentous cell giving rise to a new microcolony.

## Abbreviations

AcCoA: acetyl-coenzyme A
Ack: acetate kinase
Spc: spectinomycin
LB: Luria Bertani
FCM: flow cytometry
CDW: cell dry weight
FSC: forward scatter channel
GFP: green fluorescent protein
Pta: phosphotransacetylase
PEP: phosphoenolpyruvate
LacI: lactose repressor
PCA: principal component analysis
PB: probability binning
PI: propidium iodide
PTS: phosphoenolpyruvate:sugar phosphotransferase system
SSC: side scatter channel
TCA: tricarboxylic acid cycle
q_ac_: specific rate of acetate production
q_s_: substrate uptake rate
q_p_: recombinant product formation rate
SD: standard deviation
*gfp*: Superglo GFP gene
Y_X/S_: biomass yield from glucose
